# Naturalistic and Uncontrolled Pilot Study on the Efficacy of Vortioxetine in Binge Eating Disorder With Comorbid Depression

**DOI:** 10.3389/fpsyt.2021.635502

**Published:** 2021-03-17

**Authors:** Cristina Segura-Garcia, Marianna Rania, Elvira Anna Carbone, Renato de Filippis, Matteo Aloi, Mariarita Caroleo, Gloria Grasso, Giuseppina Calabrò, Gilda Fazia, Filippo Antonio Staltari, Antonella Falvo, Valentina Pugliese, Raffaele Gaetano, Luca Steardo, Pasquale De Fazio

**Affiliations:** ^1^Psychiatric Unit, Department of Medical and Surgical Sciences, University “Magna Graecia”, Catanzaro, Italy; ^2^Outpatient Service for Research and Treatment of Eating Disorders, University Hospital Mater Domini, Catanzaro, Italy; ^3^Psychiatric Unit, Department of Health Sciences, University “Magna Graecia”, Catanzaro, Italy

**Keywords:** vortioxetine, Binge Eating Disorder, depression, neurocognition, eating behavior, weight, treatment, efficacy

## Abstract

**Background:** Binge eating disorder (BED) is clinically relevant by virtue of the global impairment, poor quality of life, and increased overall medical morbidity. The high comorbidity with psychiatric disorders, particularly depression, has received attention as a possible mediator of the poor outcome. Further, BED and depression share cognitive dysfunctions. This naturalistic and uncontrolled pilot study aimed at evaluating the efficacy of vortioxetine (VTX) on depressive symptoms in patients with BED, secondly the efficacy in improving a broad array of executive functions, and third to explore the effect on eating behavior and body weight.

**Methods:** This pilot study involved 30 patients with BED and comorbid MDD, treated with VTX for 24 weeks. Assessments were run at baseline (*t*_0_), 4 (*t*_1_), 8 (*t*_2_), 12 (*t*_3_), and 24 (*t*_4_) weeks. Changes in depressive symptoms (HDRS and BDI), executive functions, eating behaviors (binge frequency and severity, night eating, food addiction), and body weight were estimated after treatment with VTX through GLM.

**Results:** Significant improvements emerged after treatment with VTX in: depression (HDRS *p* < 0.001; BDI *p* = 0.002) regardless the dose of VTX and first diagnosis (BED/MDD), working memory (RAVLT acquisition *p* = 0.01, delay recall *p* < 0.001, RCFT percentage of recall *p* = 0.01, and Attentional Matrices *p* = 0.05), binge days frequency (*p* < 0.001), binge eating severity (BES *p* < 0.001), night eating (*p* = 0.001), food addiction (YFAS 2.0 *p* = 0.039), and body weight (*p* = 0.039). The improvement in depressive symptoms was associated with the concurrent improvement in night eating as assessed by the I-NEQ.

**Conclusions:** VTX can be a valid therapeutic choice for patients with BED with comorbid depression in controlling the depressive symptoms, working memory, and eating behavior. Indeed, by acting on affective symptoms, neurocognitive functioning, and eating behaviors, it confirms the results already obtained with VTX in other disorders, expanding them to BED.

## Introduction

Binge eating disorder (BED) is the most frequent among eating disorders. According to recent data, 0.85–3.6% of adults (1–4) meet the DSM-5 criteria for BED (5) and women are 1.3–3 times more likely than men to be affected (6, 7).

BED is clinically relevant by virtue of its psychological burden, global impairment and poor quality of life (1, 6, 8–11), as well as increased overall medical morbidity (12–14) and access to healthcare resources.

Notwithstanding the strong association with medical conditions, some evidence suggests that medical comorbidity alone does not fully explain the overall impairment observed in individuals suffering from BED (15, 16). To this extent, the high comorbidity with psychiatric disorders, particularly depression, has received attention as a possible moderator of the poor outcome in BED (6, 15, 16).

To date, up to 65.5 and 32.8% of individuals suffering from BED self-report a lifetime prevalence of major depressive disorder (MDD) and diagnosis of persistent depression, respectively (14). Further, individuals with BED suffer from cognitive dysfunctions (i.e., poor decision making, set-shifting, inhibitory control, planning, and working memory) (17–24) and depression has been associated with pronounced cognitive dysfunctions as well (25). Even though the exact mechanisms underlying the co-occurrence and inter-relationship between depression, cognitive deficits, and BED are not fully understood, the “common-cause” model (26) may suit with this comorbidity, with cognitive inefficiency underpinning either depression and BED.

On the other hand, in line with the emotional regulation theory (27), binge eating is hypothesized to occur as a strategy to cope with negative emotional affect, in the absence of more adaptive emotional regulation strategies that are under cognitive control. Accordingly, taking into account cognitive inefficiency, depression should be considered as either a trigger or a maintaining factor associated with BED.

Given that a greater loss of control over eating has been demonstrated in BED when depressive symptoms combine with cognitive dysfunction (28), and that evidence suggests that depression and BED should be studied together rather than as distinct and independent elements (15, 29, 30), approaching simultaneously depression, cognitive disturbances, and eating behaviors may theoretically provide a more complete understanding of the clinical complexity, and potentially result in more tailored treatment strategies and improved patient's outcome (31).

The recommended first-line approach for BED includes psychological treatments, such as cognitive behavioral and interpersonal therapies (32, 33); however, about one-third of patients do not fully benefit from these treatments (34) and the reduction in binging episodes and weight loss seems to be short-lasting (35–37).

Moreover, access to such interventions may be limited by local availability and costs. Various pharmacological treatments have been used so far and have shown some effectiveness in addressing binging and weight (topiramate, naltrexone), appetite regulation (sibutramine), obsessive thoughts and compulsive behaviors (lisdexamfetamine), and reducing anxiety and affective symptoms [e.g., selective serotonin reuptake inhibitors (SSRIs), serotonin–norepinephrine reuptake inhibitors (SNRIs), and bupropion], but none of these exhibited a combined effect on all the clinical features and they showed poor tolerability and negative or no effect on cognitive symptoms (38, 39). Current treatments for BED are poor in the long term and often insufficient, therefore additional clinical trials are needed to identify effective pharmacotherapies (40).

Vortioxetine (VTX) is an innovative antidepressant with a multimodal mechanism of action that associates blocking of the reuptake of the serotonin transporter with action on multiple serotonergic receptors (5-HT3, 5-HT7, and 5-HT1D receptor antagonists, 5-HT1A receptor agonist and 5-HT1B receptor partial agonist), as well as acting on other neurotransmitters such as noradrenaline, dopamine, acetylcholine, and glutamate (41–43). VTX has a favorable tolerability profile and seems to have the pharmacodynamic properties needed to improve both depression, and cognitive functioning (44, 45).

One double-blind, placebo-controlled study (46) examined the use of VTX in eighty subjects diagnosed with BED, finding no differences in binge-eating frequency, weight, and BMI between the VTX and placebo groups. Authors argued several explanations such as insufficient length of administration of the drug (12 weeks), high attrition rates, and high placebo effect. More importantly, comorbidity with major depressive disorder was voluntary considered an exclusion criterion, and it may have contributed to recruit less severe cases and prevented to observe those cases in which the efficacy on binge-eating is secondary to the amelioration of depressive symptoms.

Based on the above, this naturalistic and uncontrolled pilot study aimed at expanding actual knowledge, by evaluating the efficacy of 24-week treatment with VTX in individuals with comorbid BED and MDD. Changes in depressive symptoms were considered primary outcome. Secondly, the study examined the efficacy of VTX in improving a broad array of executive functions. Finally, the third objective was to explore if VTX has an effect on improving eating behavior (e.g., intended as severity of eating psychopathology and dysfunctional eating behaviors) and body weight. Our hypothesis is that VTX treatment will improve not only the depressive symptoms but also the neurocognitive functioning and altered eating behaviors of individuals suffering from both BED and MDD.

## Materials and Methods

### Study Design

The study took place at the Center for Clinical Research and Treatment of Eating Disorders in Catanzaro (Italy), a tertiary multidisciplinary service providing outpatient mental health care to individuals with EDs, subsumed under the outpatient service of general psychiatry, University Hospital “Mater Domini”, Catanzaro (Italy). Individuals that consecutively referred to the outpatient unit since August 2017 to October 2019 for the treatment of BED, were screened for concurrent MDD, and considered eligible. Inclusion criteria were age 18–65 years, Binge Eating Scale (BES) (47) score > 17, Hamilton Depression Rating Scale (HDRS) (48) score > 17 and capable of answering self-report questionnaires and expressing valid consent. Participants were deemed ineligible if: diagnosis of intellectual disability from mild to severe according to DSM-5 (corresponding to IQ <70) (49); history of chronic medical illness or neurological conditions or medications affecting cognitive functioning; drug dependence and/or abuse; other severe medical comorbidities (e.g., epilepsy); pregnant, recently given birth or breastfeeding; previous diagnosis of diabetes mellitus or medications affecting glucose metabolism; known inflammatory disease; or a history of malignant disease.

The main researcher (CS-G) informed each participant individually about the aim of the study, the procedures, the voluntary nature of participation, and the management and storage of data. Participants were duly informed about the possible side effects with VTX, given the chance to leave the study at any time and asked to sign written informed consent to participate before any procedure took place. A statistical power analysis was performed with Gpower 3.1 for sample size estimation; with an alpha = 0.05 and a power = 0.80, the projected sample size needed with an effect size = 0.25 was minimum 24 participants. A total of 41 individuals were found to be eligible for the study.

The study was designed as follows: initial clinical interview, psychometric assessment and prescription of VTX were considered baseline (*t*_0_), and then psychometric assessment was rerun according to the study design timing (*t*_1_: 4 weeks; *t*_2_: 8 weeks; *t*_3:_ 12 weeks; *t*_4:_ 24 weeks) during the 24-week follow-up period (Table 1). Eleven participants were excluded from the study for the following reasons: four (3 males, 1 female) were not interested; two (1 man, 1 woman) had personal problems that prevented them from participating in the assessments; one became pregnant between *t*_2_ and *t*_3_; and four dropped out due to side effects (between *t*_0_ and *t*_2_). Data collection began in August 2017 and stopped in October 2019, when 24 weeks of data for all 30 patients had been retrieved.

**Table 1:** Evaluation schedule of assessments.

**Assessment**	**Test**	***t_**0**_***	***t_**1**_***	***t_**2**_***	***t_**3**_***	***t_**4**_***
		**baseline**	**4 weeks**	**8 weeks**	**12 weeks**	**24 weeks**
Diagnostic	Structured Clinical Interview DSM-5 (SCID-5-CV)	x				
	Binge Eating Disorder-Clinical Interview (BED-CI)	x				
Psychopathological	Hamilton Depression Rating Scale (HDRS)	x	x	x	x	x
	Beck Depression Inventory (BDI)	x	x	x	x	x
	Antidepressant Side-Effects Checklist (ASEC)	x	x	x	x	x
	Binge Eating Scale (BES)	x	x	x	x	x
	Eating Disorder Examination Questionnaire (EDE-Q)	x				x
	Food Addiction Scale 2.0 (Y-FAS 2.0)	x				x
	Night Eating Questionnaire (I-NEQ)	x				x
Neuropsychological	Stroop Color and Word Test (SCWT)	x				x
	Rey Auditory Verbal Learning Test (RAVLT)	x				x
	Rey–Osterrieth Complex Figure	x				x
	Attentional Matrices	x				x
	Iowa Gambling Task (IGT)	x				x
	Digit Symbol Substitution Test (DSST)	x				x

Participants were asked not to modify either their eating habits or physical activity during the 24 weeks of treatment in order to avoid possible confounding effects on body weight; no additional therapy (e.g., psychotherapy, pharmacotherapy) other than VTX was prescribed.

The investigation was approved by the Ethical Committee of “Regione Calabria, sezione Area Centro” (identifier: 185/D.G. 20.07.2017), in accordance with the latest version of the Declaration of Helsinki (50).

### Assessment

Well-trained psychiatrists in the ED field evaluated each patient the day of the first registration to the ED unit: patients underwent a clinical interview resulting in a DSM-5 diagnosis (mean time: 45 min), completed the HDRS with the same psychiatrist and answered the BES and the Beck Depression Inventory (BDI) (51). Diagnosis were based on the Structured Clinical Interview for DSM-5 (SCID-5-CV) (52) and the Binge Eating Disorder-Clinical Interview (BED-CI) (53). Once the diagnosis was clear, patients were invited to participate in the study. After a maximum of 1 week, the patients signed the informed consent and completed the baseline evaluation; this assessment took on average 70 min and included psychopathological (around 20 min) and neuropsychological (around 50 min) tests performed by a trained psychologist.

Psychopathological tests included the Eating Disorder Examination Questionnaire (EDE-Q) (54), the Night Eating Questionnaire (I-NEQ) (55) and the Yale Food Addiction Scale 2.0 (YFAS 2.0) (56). Neuropsychological assessment included: the Stroop Color and Word Test (SCWT; 45 s test period for each of the three sheets) (57), the Rey-Osterreith Complex Figure Test (RCFT) (58), the Iowa Gambling Task (IGT) (59), the Attentional Matrices (45 s test period for each of the three matrices) (60), the Rey Auditory Verbal Learning Test (RAVLT) (61), and the Digit Symbol Substitution Test (DSST; 45 s test period) (62). Successive administrations of psychometric tests/questionnaire followed the study design and timeline (Table 1).

Patients were measured wearing light indoor clothing and no shoes by means of a portable stadiometer and a balance scale; their height to the nearest 0.1 cm and weight to the nearest 0.1 kg were taken and their body mass index (BMI, kg/m^2^) was calculated. BMI was measured at each assessment.

Patient started therapy with VTX the day after completing all the evaluations at baseline. VTX is taken once daily without any regard to meals. The starting dosage was 5 mg/day, which was up- or down-titrated within the range of 5–20 mg/day according to efficacy (investigator's clinical judgement) and tolerability. Side effects were recorded at each assessment using the Antidepressant Side-Effects Checklist (ASEC) (63).

### Data Analysis

IBM SPSS Statistics (version 26.0) software was used for database construction and statistical analysis. Data are presented as means, standard deviations (SD), frequencies, and percentages (%). Missing data were managed with the regression imputation method, which replaces the missing data with estimated values. Instead of deleting any case that has a missing value, this approach preserves all cases by replacing the missing data with a probable value estimated by other available information. In the regression imputation, the existing variables are used to make a prediction and then the predicted value is substituted as if it were an actual obtained value. After all missing values have been replaced by this approach, the dataset was analyzed using standard techniques for complete data.

A General Linear Model (GLM) with repeated measures was used to evaluate the changes of measures across time. Possible confounding variables were included in the analysis as covariates (i.e., depressive symptomatology improvement, BED/MDD as first diagnosis). Eta-squared (η^2^) was used as a measure of the side effects of the GLM, considering values of 0.01, 0.06, and 0.14 as indicating small, medium, and large effects, respectively. The IGT, RCFT, RAVLT, SCWT, DSST, and Attentional Matrices were not normally distributed according to the Kolmogorov-Smirnov test, therefore the Wilcoxon test was run to analyse the differences between *t*_0_ and *t*_4_; r was calculated as a measure of the effect size of the Wilcoxon test, considering values of 0.10–0.29, 0.30–0.49, and ≥ 0.5 to be indicative of small, moderate, and large effects, respectively. McNemar non-parametric test was used to compare the proportion of patients positive to YFAS 2.0 before and after treatment. Considering the exploratory and naturalistic approach, *p* < 0.05 was considered to be significant. The Benjamini-Hochberg procedure (64, 65) was used to correct for multiple comparisons.

## Results

A total of 30 from 35 participants (85.7%) completed the study. Causes of dropouts were: one patient became pregnant between *t*_2_ and *t*_3_, three patients had moderate to severe nausea (between *t*_0_ and *t*_1_), and one patient suffered unbearable vomiting (between *t*_2_ and *t*_3_).

The mean daily dose of VTX taken by completers was 15.8 ± 5.6 mg (median = 20 mg) and the mean dose among dropouts was 12.6 ± 5.6 mg (median = 15 mg). Table 2 shows the main demographics. Women, high school education level and people not occupationally active were the most frequent parameters. Twenty participants (66.7%) referred an earlier onset of BED with respect to MDD (Table 2).

**Table 2:** Sample description.

		**fr**	**%**
Age^*^		42	12.9
Age at onset^*^	BED	30.0	11.8
	MDD	36.3	13.8
First diagnosis	BED	20	66.7
	MDD	8	26.7
	Simultaneous	2	6.7
Sex	Male	4	13
	Female	26	87
Civil status	Single	12	40
	Married	16	53
	Divorced	2	7
Occupation	Student	2	7
	Housewife	10	33
	Worker	11	37
	Unemployed	6	20
	Retired	1	3
Education	Middle school	9	30
	High school	18	60
	University	3	10

* *presented as means and standard deviations*.

**Figure 1:**
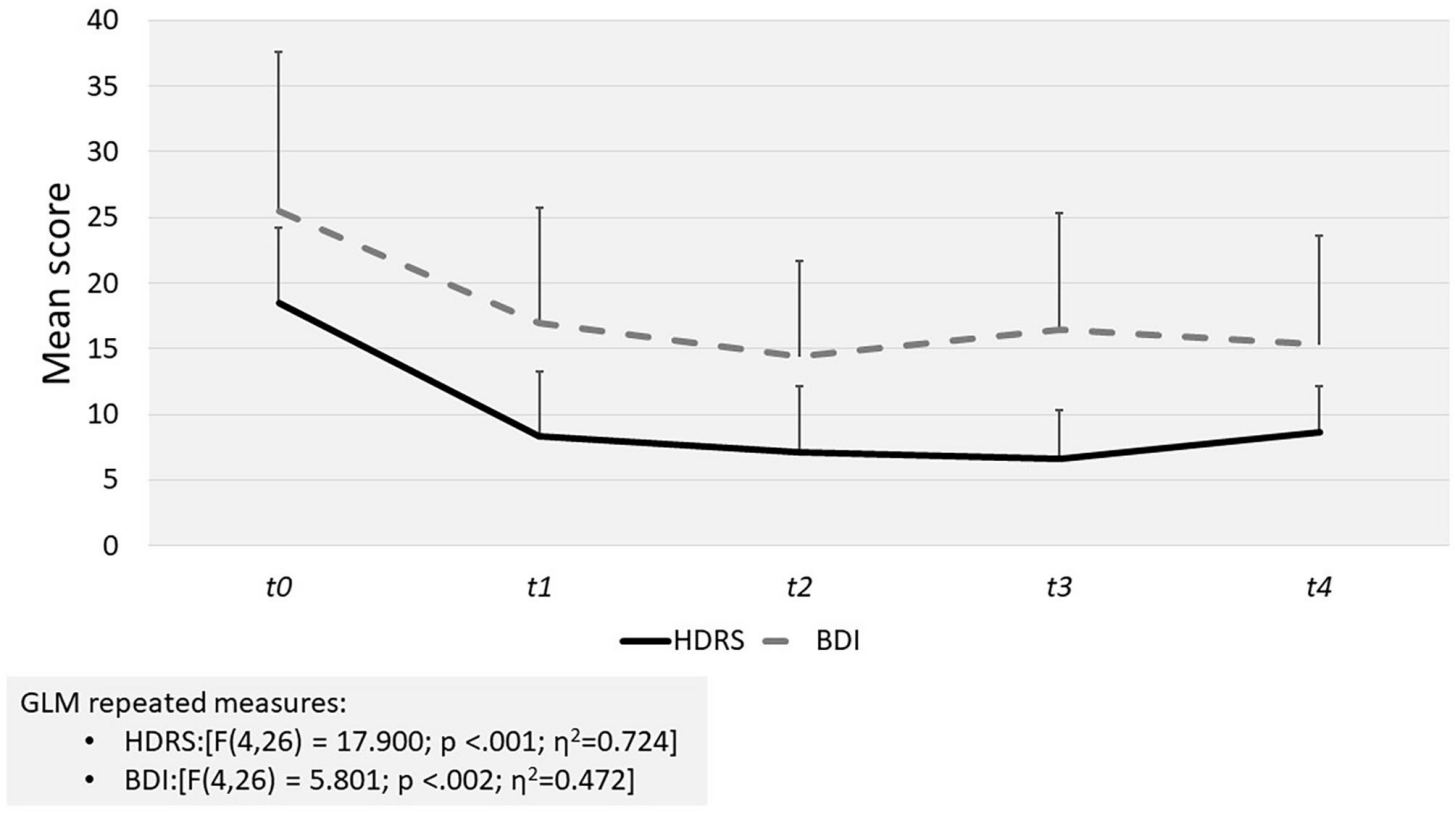
Results of GLM repeated measures for self-report (BDI) and clinician rated (HDRS) depression tests scores.

The primary endpoint was the change in HDRS and BDI scores from baseline. Depression significantly reduced overtime, as demonstrated by both the HDRS (*p* < 0.001) and the BDI (*p* = 0.002) scores (Figure 1). Large effect sizes were evident in all cases. Differences remained significant when controlling for VTX dosage and first diagnosis (i.e., BED/MDD onset). The rates of remission were respectively, 36.7% and 23.3 for HDRS (≤7) and BDI (≤9).

The secondary endpoint was the change in neurocognition scores (Table 3). Significant improvement was evident in the RCFT percentage of recall (*p* = 0.01), the RAVLT acquisition (*p* = 0.01), and delay recall (*p* < 0.001) and Attentional Matrices (*p* = 0.05) after treatment, otherwise an improvement in the RCFT order (*p* = 0.069) was observed. No differences related to the IGT, SCWT, and DSST scores were found.

**Table 3:** Neurocognitive testing: Wilcoxon test.

		***t_**0**_***	***t_**4**_***	**Wilcoxon (Z value)**	***p***	***r***
RAVLT^*^	Acquisition	47.4	53.6	−2.757	**0.006**	0.348
	Delay recall	11.3	12.8	−2.588	**0.010**	0.270
Attentional Matrices^*^		53.2	57.0	2.236	**0.025**	0.130
IGT net score		−10.2	−1.7	1.473	0.141	
SCWT		1.0	2.8	1.594	0.111	
DSST		26.8	27.2	0.547	0.584	
RCFT^*^	Accuracy	34.7	35.1	1.138	0.255	
	Order	1.6	2.0	1.882	0.069	
	Style	1.2	1.3	0.797	0.425	
	Central coherence index	1.1	1.3	1.183	0.287	
	Organizational strategies	4.2	4.5	0.654	0.513	
	Recall percentage^*^	63.4	72.1	2.509	**0.012**	0.327

*RAVLT, Rey Auditory Verbal Learning Test; IGT, Iowa Gambling Task; SCWT, Stroop Color and Word Test; DSST, Digit Symbol Substitution Test; RCFT, Rey-Osterreith Complex Figure*.

* *In bold significant results after using the Benjiamini-Hochberg procedure to correct for multiple comparison. Effect size (r) only for significant results*.

The third endpoint were the changes in eating behavior and body weight (Table 4). Mean BMI decreased after treatment (*p* = 0.039) but the reduction was not significant after applying the Benjamini-Hochberg procedure for multiple comparison (*p* < 0.03); instead, significant reductions in the BES (*p* < 0.001), and I-NEQ (*p* = 0.001) scores were evident; the EDE-Q did not change. The percentage of patients meeting the criteria for food addiction (YFAS 2.0) was significantly lower after treatment (*t*_0_ = 70% vs. *t*_4_ = 46.7%; McNemar's test = 4.000; *p* = 0.039). The average frequency of weekly binge days significantly decreased from *t*_0_ = 4.9 ± 1.9 days a week to *t*_4_ = 2.7 ± 2.4 days a week (*p* < 0.001). After controlling for changes in depression and first diagnosis (i.e., BED or MDD), the reduction in BES and the weekly binge days remained significant; on the other hand, a significant effect of changes in depression on I-NEQ score emerged (*p* = 0.009).

**Table 4:** Body Mass Index and eating behavior: GLM repeated measures.

	***t_**0**_***	***t_**1**_***	***t_**2**_***	***t_**3**_***	***t_**4**_***	***F***	**P**	**η^2^**
BMI	38.8	38.4	35.9	34.8	33.6	time: *F*(4,25) = 2.954	0.039	0.312
Weekly binge days^*^	4.9				2.7	time: *F*(1,29) = 45.673	**<0.001**	0.612
BES^*^	22.1	17.0	15.6	14.2	13.3	time: *F*(4,26) = 7.771	**<0.001**	0.543
EDE-Q	3.4				3.2	time: *F*(1,29) = 0.392	0.536	
NEQ^*^	19.1				15.5	time: *F*(1,29) = 12.312	**0.001**	0.298

*BMI, body mass index; BES, Binge Eating Scale; EDE-Q, Eating Disorder Examination Questionnaire; NEQ, Night Eating Questionnaire*.

* *In bold significant results after using the Benjiamini-Hochberg procedure to correct for multiple comparison. Effect size (η^2^) only for significant results*.

## Discussion

This study aimed at evaluating the efficacy of VTX on depressive symptoms, executive functions, eating behavior, and body weight in individuals with comorbid BED and MDD. BED most likely involves abnormalities in three domains regulating eating behavior: the ancestral 'peripheral-hypothalamic' feedback system, the reward system, and the top-down control circuitry (66). Thus, on a neurochemical level, BED may be related to the dysfunction of the serotonergic, dopaminergic, noradrenergic, and glutamatergic pathways (67). This tri-monoaminergic neurotransmitter system, with the recent focusing on glutamate, is the pathophysiology basis of depression as well, and still leads existing treatment options (68, 69). Therefore, a medication to target binge eating, as well as BED in comorbidity with depression, needs to be multi-modal in terms of its pharmacology, and VTX pharmacological profile theoretically addresses all these needs (70).

The main finding of the study was an improvement of depressive symptoms, working memory and disordered eating behavior following treatment with VTX. To the best of our knowledge, this is the first study to show this improvement.

### Efficacy of VTX on Depression

VTX has already confirmed its efficacy in major depressive episode, in adults and elderly patients, with specific effect on cognitive impairment and physical symptoms (71–77), nonetheless, there are still no studies demonstrating its effectiveness in patients suffering from depression and BED.

As above mentioned, only one study examined the use of VTX in BED, and voluntarily excluded comorbidity with MDD in order “to avoid the confounding variable in a small sample of depression possibly worsening the binge behavior” (46). However, the role of depressive symptoms in BED is still debated and current evidence suggests that the two phenomena should be studied together rather than as distinct and independent elements (15, 29, 30).

In this regard, our results showed an improvement in both self-report and clinician-rated depression, independent of which disorder came first (i.e., BED or MDD). Thus, the reduction of depressive symptoms among patients with MDD and BED is comparable to that reported in the literature for other patients with MDD without this comorbidity treated with VTX (78–80).

The low number of dropouts confirms the good tolerability of VTX. Common side effects were similar to those previously reported (45, 81–83), quite easy to manage and not life-threatening. The favorable tolerability profile of VTX can promote adherence to treatment, which is an important factor for therapeutic success.

### Efficacy of VTX on Neurocognition

Working memory significantly improved within the treatment. Working memory is crucial for several cognitive processes in daily life (84, 85), and depression affects both the phonological loop and visuospatial sketch pad of working memory (86).

Our results put in evidence the improvement of verbal (i.e., the RAVLT) and visual (i.e., the RCFT recall) memory, although the DSST score was unchanged after treatment. Other studies previously examined the efficacy of VTX on the RAVLT in patients with depression and reported significant improvement in the verbal memory domain (75, 87, 88).

This is the first study reporting a positive change in visual memory using the RCFT (i.e., RCFT recall) in the context of the efficacy of VTX. These results should be read in light of the concurrent observed improvement in the attentional domain (i.e., Attentional Matrices). It can be speculated that there is an interplay between attentional capabilities and further working memory performance. This is very important because previous studies demonstrated visuospatial memory deficits (89) and impaired performance on the RCFT recall due to poor visual attention (17, 19) among BED patients.

In our study, slight but not significant changes were evident for the IGT, SCWT, and DSST after treatment. The difference between ours and others' studies deserves further explanation. The first difference regards the sample: ours are patients with comorbid BED and MDD; instead, in most studies (75, 87, 90) patients had only the diagnosis of MDD. Other researchers reported significant differences in these tests after treatment with VTX (91); nonetheless, it is known that positive outcomes of treatment on cognitive functioning are bigger among those patients occupationally active independently of the improvement of depression (90), whilst our different results could be explained not only by the smaller sample size but also in that people who did not work were overrepresented in our sample. Importantly, previous protocols used a longer administration time for the DSST (i.e., 90–120 s vs. 45 of our protocol) enabling the comparison with present results (87, 88).

Notwithstanding the improvement in different cognitive functions, it is not possible to ascertain if this effect remained over and beyond the improvement in depression as previous studies reported in patients with MDD (75, 92–94). Recently, the potential role of depression on neuropsychological performances of patients with eating disorders, in particular patients with Anorexia Nervosa (AN) (95, 96) and Bulimia Nervosa (BN) (97, 98) have been investigated. More specifically, the association between poor performance on the neuropsychological tests and the diagnosis of AN weakened after adjusting for depression, and the authors argued that cognitive impairment may be overestimated if depression is not taken into account. Contrarily to AN, who still perform worse than HC after controlling for depressive symptoms, depression explains the whole cognitive impairment among patients with BN (98). Summing up, although previous studies demonstrated a direct effect of VTX on cognitive improvement independent of changes in depressive symptoms (75, 92–94), future studies should ascertain the unique effect of VTX on the neurocognitive profile of patients with comorbid BED and MDD.

### Efficacy of VTX on BMI and Eating Disorder

The peculiar multimodal mechanism of action of VTX has previously been demonstrated not to affect the body weight of patients in the short or long term (41, 83), even though a clinically significant weight reduction was observed in previous clinical studies (45, 99, 100). A nearly statistically significant weight reduction (overall, 5 points of BMI) after 24 weeks of treatment with VTX was evident in our sample. As the participants were patients with BED and we controlled weight loss after a longer follow-up, our results are not comparable with the above-mentioned studies.

Present results confirm that binge eating frequency reduced across the time under treatment with VTX, supporting Grant et al. (46) findings. On the contrary, we additionally found a significant improvement of BES score after 24 weeks, probably due to the longer duration of our trial. A modest efficacy of antidepressants in the short-term reduction of binge episodes in BED, with no significant impact on weight, emerged in a recent meta-analysis but the results in long-term studies are disappointing (101). SSRI antidepressants seem relatively well-tolerated but some patients experienced side effects (39). Moreover, weight gain is a common adverse event associated with some SSRIs and SNRIs (102–104). Studies with other classes of antidepressants, such as SNRIs (105) and bupropion (106), have shown discordant results on binge improvement or weight loss in BED. More recently, the association of naltrexone/bupropion (extended release) has proved to be effective in reducing BMI and altered eating behaviors in obese patients with BED in a 16-week study (107); however, the authors did not find changes in depressive symptoms. Herein, although depressive symptoms improved, they did not affect the improvement in binge eating outcomes, that most likely improved by virtue of the multimodal targeted action of VTX on the serotoninergic pathways.

The NEQ scores also decreased after treatment with VTX. Of note, this improvement was associated with the concurrent improvement in depressive symptoms. Previous studies supported an association between night eating and both depressed mood (108) and BED (109). Others pointed out that nocturnal hyperphagia or binging behaviors, and the consequential weight gain in these patients may reflect an alteration in the serotonin system for regulating appetite and food intake (110). Present results suggest a direct mediation effect of depression improvement in night eating behavior.

Food addiction is uniquely associated with BED, with prevalence estimates of food addiction up to 92% in individuals with BED (111, 112). Given the high co-occurrence and symptoms overlap (e.g., loss of control over eating, access to food despite negative consequences, failed attempts to quit) (113), BED and food addiction may be hard to disentangle. Furthermore, food addiction positively correlates with depressive symptoms in non-clinical adolescent sample (114) and in adolescents with obesity seeking weight-loss treatment (115), and the more severe depressive symptoms, the more likely the odds of having severe food addiction is (OR = 13.2, for severe depression; OR = 15.6 for extremely severe depression) (116).

In present study, the percentage of patients with MDD and BED fulfilling diagnostic threshold for food addiction significantly decreased after treatment. To date, there is no evidence of pharmacological interventions targeting food addiction. Given that some of the major symptoms of food addiction are thought to be under the serotoninergic control (e.g., craving, impulsivity) (117, 118), this result could be explained with the unique effect of VTX. However, the mediating effect of the improvement in both binge eating and depression cannot be excluded, then this result should be taken cautiously, and need to be replicated.

General eating psychopathology did not improve within the treatment with VTX. A possible explanation may be that EDE-Q total score mostly assesses cognitive domains related to eating disorders, such as eating concern, shape concern, and weight concern, and only considers restriction among eating behaviors. Accordingly, we can speculate that the EDE-Q total score may not be so accurate in detecting short-term improvements in patients with BED, especially considering that higher levels of restriction after treatment might be expected.

### Practical Considerations

BED and MDD may share underpinning dysfunctions of the serotonergic, dopaminergic, noradrenergic, and glutamatergic pathways (66). Therefore, a multimodal medication in terms of its pharmacology, and the pharmacological profile such as VTX, may be theoretically an effective option to target the comorbidity between BED and MDD (70).

### Strengths and Limitations

Study strengths include rigid inclusion criteria, which avoided the introduction of unmeasured bias (e.g., other treatments), and strict analysis controlling for multiple testing. The selection of patients with concurrent BED and MDD let us consider a subsample with more severe general psychopathology, which may respond better in terms of eating psychopathology thanks to the treatment for depression itself. In fact, excluding these patients from the evaluation could introduce severity bias, and a failure in detecting those improvements in eating psychopathology accounted by concurrent depressive symptoms. Therefore, either self-report and clinician rated depression severity was evaluated, giving more consistency and completeness to results. HDRS is considered the gold standard for evaluating depression in trials (119, 120), showing good sensitivity in capturing the effectiveness of pharmacological treatments (121, 122).

Notwithstanding the efficacy of VTX on BED and concurrent depression, present results should be read in light of some limitations. The naturalistic uncontrolled design, specifically the lack of a control group, prevented to conclude if the efficacy of VTX is comparable to other treatments currently used for BED (pharmacological or psychotherapy) or placebo. The follow-up was large enough to catch psychopathological and anthropometrical improvements (titration time plus latency time before efficacy of VTX), however we cannot support this improvement is maintained in the long-term. Failure in outcomes in the long-term is one of the most critical points in the existing literature on BED, so further studies, with larger follow-up are encouraged to confirm present results. Lastly, we selected seeking-active treatment patients with BED and concurrent MDD, given the very high comorbidity of these two disorders in the real-life clinical setting. However, these patients may suffer from more severe psychopathology, accordingly present results cannot be generalized to patients with BED without the comorbidity or to patients that actually do not seek treatment or to less severe cases.

### Conclusion

In summary, this longitudinal open-label study shows that VTX may be a valid therapeutic choice for patients with BED with comorbid depression in controlling their depressed mood, their working memory and their eating behavior. Indeed, by acting on affective symptoms, neurocognitive functioning and eating behaviors, it confirms some results already obtained with VTX in other disorders, expanding them to BED. Finally, a better characterization of patients with BED that takes into account other psychiatric comorbidities, especially mood disorders, seems necessary.

## Data Availability Statement

The raw data supporting the conclusions of this article will be made available by the authors, without undue reservation.

## Ethics Statement

The studies involving human participants were reviewed and approved by Ethical Committee of Regione Calabria, sezione Area Centro (identifier: 185/D.G. 20.07.2017). The patients/participants provided their written informed consent to participate in this study.

## Author Contributions

CS-G and PDF contributed to the conception and design of the study. MC, GC, GF, FS, AF, and GG organized the database. CS-G performed the statistical analysis. RdF, MA, MR, EC, and LS wrote the first draft of the manuscript. VP and RG critically revised the manuscript. All authors contributed to manuscript revision, read, and approved the submitted version.

## Conflict of Interest

The authors declare that the research was conducted in the absence of any commercial or financial relationships that could be construed as a potential conflict of interest.

## Abbreviations

ASEC: Antidepressant Side-Effects Checklist
BDI: Beck Depression Inventory
BED: Binge eating disorder
BED-CI: Binge Eating Disorder-Clinical Interview
BES: Binge Eating Scale
BMI: body mass index
DSST: Digit Symbol Substitution Test
EDE-Q: Eating Disorder Examination Questionnaire
GLM: General Linear Model
HDRS: Hamilton Depression Rating Scale
IGT: Iowa Gambling Task
NEQ: Night Eating Questionnaire
RAVLT: Rey Auditory Verbal Learning Test
RCFT: Rey-Osterreith Complex Figure Test
SCID-5-CV: Structured Clinical Interview for DSM-5
SCWT: Stroop Color and Word Test
SD: standard deviation
SNRIs: serotonin–norepinephrine reuptake inhibitors
SSRIs: selective serotonin reuptake inhibitors
VTX: vortioxetine
YFAS 2.0: Yale Food Addiction Scale 2.0.
